# Is Malaria a Land or Water Disease?

**Published:** 1897-06

**Authors:** 


### EDITORIAL DEPARTMENT.

F. E. DANIEL, M. D., Editor.
S. E. HUDSON, M. D., Managing Editor.
A. J. SMITH. M. D., Galveston, Associate Editor.

EDITORIAL STAFF:

PROF. J. E. THOMPSON, M. D., Texas Medical College, Galveston; Surgery.
PROF. WM. KEILLER, M. D., Texas Medical College, Galveston; Obstetrics and Gynecology.
PROF. DAVID CERNA, M. D., Texas Medical College, Galveston; Therapeutics.
PROF. A. J. SMITH, M. D., Texas Medical College, Galveston; Medicine.
DR. R. H. L. BIBB, City of Mexico; Foreign Correspondent.

Published Monthly at Austin, Texas, by Drs. Daniel and Hudson. Subscription price $2.00 a year in advance.

Eastern Agents: The Monihan-Fairchild Special Agency, 24 Park Place, New York City.

Official organ of the West Texas Medical Association, the Houston District Medical Association, the Austin District Medical Society, Brazos Valley Medical Association, the Galveston County Medical Society, and several others

### IS MALARIA A LAND OR WATER DISEASE?

Dr. J. E. Gardiner in this issue makes an argument that is apparently unanswerable, to show that malaria is exclusively a water product, and he presents *data* that is seemingly convincing. Dr. Gardiner is a good observer, and has made a painstaking record of his observations, and his opportunities for observation, too, have been good, his *data* covering a wide scope of country; hence, his opinion is entitled to great weight.

It is but another illustration of the old saying that "Doctors will differ." We cite in support of this assertion, Dr. Shrady, in the New York *Medical Record* of recent date, after reviewing all the evidence at hand, pro and con, states his opinion emphatically that malaria is of soil origin, and is atmospheric, and while water may become and does become impregnated with the poison, and hence may produce sickness, it is yet possible for a person to drink pure water and still suffer from malaria if he reside in a malarial *atmosphere.*

Dr. Bussey, of Washington City, in a lecture recently to the graduates of the Army Medical College, stated his conviction that malaria is communicated solely through the medium of the atmosphere; alleging, in support of his position, that the plasmodium malariæ has never been found in water. Indeed, it has never been found anywhere, outside of the blood of man, unless it be in that of the pigeon, as has been claimed. It appears to be universally admitted by both parties to the controversy that the poison is generated by the action of the heat and moisture upon organic matter; still the germ known as the *plasmodium malariæ* has never been found in any decaying vegetable matter. It is thought that the poison, whatever may be its original form and nature, does not enter the blood as plasmodia, but that it attains that form in the blood by evolution from a more rudimentary state.

The question is like that of the shield, which, to one observer was golden and to another argent. When looked at on both sides, it was found to be both golden and argent. So with malaria. Equally as strong proof of its exclusive atmospheric origin can be and is produced. The truth lies, doubtless, between the two extremes; and when the controversy is finally settled we believe it will be found that malaria is communicated to man through both media—atmosphere and water. The JOURNAL invites a discussion of the question, if any of its readers feel disposed to question Dr. Gardiner's conclusions.

